# The Toxins of Insanity1Read before the Buffalo Medical Club, November, 1902.

**Published:** 1903-04

**Authors:** James Wright Putnam

**Affiliations:** Buffalo, N. Y., Professor of Nervous Diseases, University of Buffalo; President of the American Neurological Association, 525 Delaware Avenue.


### BUFFALO MEDICAL JOURNAL.

VOL. XLII.—LVIII. APRIL, 1903. No. 9.

### ORIGINAL COMMUNICATIONS.

### The Toxins of Insanity.[1]

BY JAMES WRIGHT PUTNAM, M. D., Buffalo, N. Y.,

Professor of Nervous Diseases, University of Buffalo; President of the American Neurological Association.

THE clinician in the daily round of practice comes in contact with diseases which so change physiologic processes that new products are formed. These products act upon nerve tissues as poisons and, according to the individual's heredity or personal tendency, he is afflicted with some form of disturbance of the nervous system. Prominent among the diseases which may so generate or produce poisons are Bright's disease and diabetes mellitus.

We are all familiar with the facts. Many cases of Bright's disease show headache, insomnia, interference with intellectual processes. The ophthalmologists meet with the choked disc or albuminuric retinitis, uremic convulsions, coma and paralyses, which may be monoplegias or hemiplegias. All may occur as a result of the retention of various tissue poisons in the blood, which should have been eliminated by the kidneys. To diagnosticate uremic coma, is not as easy as some of the textbooks would lead us to suppose. Usually the coma is more profound after cerebral apoplexy and after an epileptic convulsion. The pupils in the coma of Bright's disease may be normal, contracted or dilated; they are rarely unequal. The temperature is usually subnormal, which is not the case in coma from apoplexy, or in the coma of status epilepticus.

The blood, according to Von Jaksch, always shows excess of acid, so that given a case of coma with excess of acid, low tem-

1. Read before the Buffalo Medical Club, November, 1902.

perature and retinitis, we have a right to make a diagnosis of Bright's disease or uremic coma. In place of coma, chronic nephritis may terminate in muttering delirium.

Insanity of various types has been observed in both the acute and the chronic varieties of Bright's disease. Insanity is much more frequent in the cases with contracted kidney than it is with the large white kidney. The insanity may appear when the presence of albumin is only indicated as a trace in the urine. The most commonly observed form is the confusional insanity, with hallucinations and with restlessness. So important a fact is this that Berkley says: "The presence of confusional insanity nearly always indicates a toxemia from some source and it behooves the physician to make frequent and accurate examinations of the urine in all suspicious cases in order to avoid unfortunate mistakes in diagnosis." We may also have simple melancholia, or a more active melancholia with distinct delusions of persecution, or we may meet with mania and dementia.

In diabetes, we find an evidence of a tendency to nervous tissue changes in a variety of symptoms. The oculist meets ophthalmoplegias, myosis, optic nerve atrophy, retinitis, and retinal hemorrhages. The general practitioner encounters diabetic apoplexy, headache, migraine, neuritis and attacks of vertigo. The alienist will meet with varied forms of insanity.

It is to be stated, however, that diabetes rarely causes insanity in individuals with a good heredity. In individuals predisposed to insanity the poison from glycosuria is apt to bring on mental changes. Melancholia which improves as the diabetes improves has been observed. Maniacal disturbances, in which there is great activity has been described. On the other hand, no less an authority than Savage, who was for years at the head of Bethlehem, more popularly known as Bedlam, in England, says: "I know of no direct relation between diabetes and insanity."

Berkley refers to the fact that in diabetes we may have a grouping of symptoms which resembles paretic dementia, viz.: the pseudo-paresis diabetica.

The first period is marked by headache and vertigo. This is followed by loss of interest in affairs, loss of memory. This is succeeded by a period of excitement with shifting delusions of grandeur. These persist for a time to be succeeded by tremor in the muscles of the face, changes in speech, changes in patellar and pupillary reflexes, and terminates in dementia. Acetone is probably the poison responsible for the final coma.

With the intoxication insanities dependent upon a disordered alimentary canal, we have all had some experience, I came near saying personal experience, for few of us have escaped the mental symptoms known as apathy and disinclination to work, accompanied by some irritability, and associated with this some headache. Such symptoms being temporary in character, we have not classified as insanity, but have called them indigestion and constipation, and have cured them by blue pills, salines, abstinence from food and by enforced exercise. But there are cases on record of actual insanities of true intestinal origin. One was by Solders, recorded in the *Jahrbuch für Psychologie*, Bd. xvii.

A woman, 40 years old, had been constipated for six days, then became suddenly insane, showing a typical picture of confusional mania with excitement. All the symptoms disappeared promptly after the bowel was unloaded.

Berkley records a case of a man 70 years old who after constipation lasting one week, had an intense headache, this was followed by clouding of mental faculties and the hallucination of a head with a long grey beard beside his own head. Arguments were of no avail. The hallucination persisted until under lavage and saline cathartics he recovered in twenty-four hours and did not relapse.

The chemical examination of the urine shows two kinds of autotoxins from the intestinal tract. Acetone has been found in large quantities, and also diacetic and oxybutyric acid in the urine of patients with mental symptoms.

The decomposition of albumin in the bowel shows itself in the urine as ethereal sulphates, as indican, or indoxyl-potassium sulphate, and as skatol or skatoxyl-potassium sulphate. The indican appears as a result of fermentation in the small intestine and is usually much increased by constipation. The presence of skatol is generally an indication of decomposition of matter in the large intestine. There is no difference in the symptomatology of the insanities produced by the toxins of the ethereal sulphates, or of acetone. The onset is usually gradual. There is dull, heavy headache, hypochondriasis, mental confusion, and sometimes stupor. There is usually no fever.

Berkley says through its whole course the disease is usually afebrile and there are no remissions. In these respects it differs from acute delirium. Like acute delirium there may be great motor agitation with rapid loss of strength.

Again, in autointoxication manias, we may have a folie circulaire. There will be ten to fourteen days of hallucination, with excitement; this is succeeded by a longer period of inertia, sometimes amounting to stupor; then a gradual return to normal, active mental life in the course of about two or three months. Recovery may be complete or the cycle of mental phenomena may be repeated. Autopsies in such cases have been made. They have revealed changes in the mucous membrane of the intestines, constipation, and parenchymatous degeneration in the kidneys and liver.

General hyperemia of the brain and membranes with edema has been reported. In the insanities produced by alterations in the secretions of the thyroid, we have a most interesting subject. Those of us who have seen much of exophthalmic goitre must have noted mental changes in a certain percentage of cases. There is in the hypertrophied gland a change in the thyroid secretion either in quantity or in quality, which exerts a powerful influence upon the nervous system. This influence is shown in the rapid heart beat, in the neurasthenia, hysteria and in the various insanities.

Berkley says that the attendant psychosis of exophthalmic goitre is usually of the maniacal type, sometimes characterised by morbid impulses, insane actions and insistent ideas.

My own experience is singularly different from this. I have never seen mania or anything approaching it in Graves's disease, but I have observed profound mental depression, marked by disinclination to read, a wish to be left alone by friends, and a tendency to frequent attacks of weeping and suicidal ideas.

The course of insanity in three of Berkley's patients was very gradual, lasting for several years and all passing into terminal dementia.

Gowers says of the insanities of exophthalmic goitre: slight mental change, irritability, depression and restlessness are common, but in the cases amounting to actual insanity, which often begin with hallucinations, the form varies. "It may be melancholia or simple mania, or a general paralysis of the insane. Of these, mania is the most common and may be the cause of death. It sometimes lapses into chronic insanity."

Again, in patients suffering from exophthalmic goitre, who have no mental symptoms, operations removing a part or the whole of the thyroid gland have been followed by a post-operative myxedema. This is usually accompanied by marked mental changes in which periodic mania and circular insanities are common. Other cases of myxedematous insanity become stupid, feeble and untidy and show every sign of profound poison. It is in this type of insanity that we see the striking result of thyroid therapy. Patients with proper doses of thyroid extract improve with astonishing rapidity in mental and physical symptoms.

While upon this subject of myxedematous insanity, I shall take the liberty of digressing enough to quote from Osler's paper on Sporadic Cretinism in America,—his summing up of the character of the changes in myxedematous cretins under the thyroid treatment.

(*a*) Bodily—loss in weight, due to disappearance of the myxedematous condition and of the fat, is noticed within a month or six weeks after the commencement of the treatment. The face becomes thinner, the palpebral orifices wider, the puffiness disappears from about the eyes, the flabby supraclavicular folds melt away, the projecting abdomen diminishes in girth, and the child's figure becomes more shapely. Several of the photographs illustrate this in an interesting manner. This change is much more striking in young children of from three to six or eight years, but it is also well seen in the older patients. Nothing could be more remarkable than the change in the features in Dr. Carson's case, and even in Dr. Sinkler's case, aged 30 years, the change, as shown in the photograph, is most evident. The expression of the face is altered by the recession of the tongue, and in many instances the drooling ceases as the mouth is kept closed; this relieves in great part the idiotic expression.

Among the constructive and progressive alterations may be mentioned the loss of the waxy pallor of the skin, which become softer and much more natural-looking. The hair, too, changes and becomes more abundant and finer. Several writers have referred very particularly to this remarkable change in the skin and hair, as though there had been a complete substitution of the old by a new skin and hair. In very young children teething proceeds rapidly; in older subjects, if the second dentition has not begun, the milk-teeth are shed and the permanent ones develop rapidly. No change is so remarkable as the increase in stature, as Dr. John Thomson remarks, "the natural impulses of growth, which were in abeyance in the thyroidless condition, are let loose."

In my first case the little girl grew four inches in a year. Among the most remarkable in the collected series are the following: Dr. Friend's case gained eleven and three quarter inches in one year and ten months; Dr. Vincke's case gained nine inches in one year and seven months, and Dr. Noyes's case gained eight inches in four and a half months, and Dr. Edwin F. Wilson's case gained seven inches in six months.

(*b*) Mental change—even within a couple of months the alteration in the mental condition is noticed. At any rate, the patients look much brighter and the face is not absolutely expressionless. As a rule, the younger the case the more marked is the mental change. Young cretins who have not learned to speak a word soon begin to talk in their play. In children between six and ten, the effects are even more remarkable, and with the loss of the myxedematous condition there is a corresponding awakening of the mental faculties. In older patients the treatment is not so efficacious. In case II. of Osler's series, the girl aged 19 years did not seem to be very much benefited, although it is true the treatment was abandoned by the mother after a short time. In other instances, as in Dr. Sinkler's case, the mental condition improved very much, even though the patient was over 30 years old.

Insanity of pregnancy is rather rare, for the mental changes observed during gestation do not often amount to insanity In those women affected with the albuminuria or glycosuria of pregnancy, there have been several cases of melancholia or mania reported. These cases have been attributed to the toxins of the ammonium series. It is especially the postparturient insanity that is of definite septic origin. The postpartum insanities are divided by Berkley into (*a*) those cases in which infection comes from some portion of the genital tract, or in a few instances from the mammæ; (*b*) the toxin from the accumulation in the blood and tissues of carbamate of ammonium and chemical products of incomplete tissue metamorphosis, producing irritation of the nervous system.

In a few cases septic infection can be excluded. This last class it is not our province to discuss.

The first class, induced by infection through the genital tract, is the typical puerperal insanity. All recent writers are united in the opinion that infection is the most frequent cause.

In Johns Hopkins, Prof. Whitridge Williams reported two cases of puerperal mania in which cultures taken from the interior of the uterus showed the streptococcus pyogenes. Berkley had one case of puerperal mania in which cultures from the blood and from the vaginal secretions showed streptococci. Clouston also had a case of puerperal mania which showed at the post-mortem pus on the peritoneal surface of the uterus. The mucous membrane of the uterus showed purulent material.

There were deposits of pus all through the uterine wall. The most frequent primary seat of the infection is presumably the placental site, as the open vessels afford an easy and ready means for conveying infection from unclean exploring hands or instruments, but nature is kind and wonderfully protecting, for according to McLeod, 1 in 1950 parturient women was affected in England with puerperal insanity as gathered from the statistics of hospitals from 1878-1882. From all tables published the records show that one woman in 616 deliveries becomes insane; yet it is probable that more than 1 in 616 run the risk of infection.

Beside the placental site contagion may be traced to lacerated perineums, bruising and contusions of the vagina. In considering the subject of infection it is necessary to refer to a paper of Dr. Hobbs, superintendent of the asylum for the insane at London, Ont. He classifies the septic insanities of the puerperium under three heads: (1) puerperal insanity with little or no local lesion caused by septic infection. These insanities he believes occur probably from absorption into the circulation of the toxins of an infected clot, or by the absorption of the saprophyte germ which finds lodgment in the detritus of a puerperal uterus. The majority of these cases are of short duration and recover at home; (2) puerperal insanity, complicated by gross local lesion, the result of sepsis. These cases are more serious as the local inflammatory lesion keeps up a continued supply of the virus, so that the majority of the patients do not recover under ordinary treatment; (3) he calls post-puerperal insanity induced by pelvic disease, which is the result of septic infection. These cases are the ones in which insanity develops in 6 to 8 months after the accouchement.

Of the last class he says in the past four and one-half years 80 cases were admitted to the asylum with inflammatory disease of the pelvic organs, brought about by septic invasion. All of these 80 cases had chronic metritis, 42 had diseased cervices, 33 had retrodisplacements, 19 had lacerations of the perineum, 11 had inflammatory, tubal or ovarian disease, 3 had fibroids, 1 a deep seated rectal fistula. Upon suitable surgical treatment, 36 were cured mentally, 20 more were improved, 24 were unimproved. He concludes with a strong plea for the prevention of septic invasion. In the discussion of this paper, Dr. Carlos Macdonald placed himself on record as believing that a considerable proportion of puerperal insanities were due to sepsis. Savage, of

England, is as yet unconvinced of the septic origin of many puerperal insanities.

There still remains for our consideration the psychoses of the different fevers. Of these typhoid fever, influenza, pneumonia and scarlet fever, according to Berkley, rank first in importance. Regis, in his book, holds that scarlet fever, measles and diphtheria very rarely produce insanity. He places variola as the eruptive fever most frequently complicated by insanity. Regis states that in convalescence from variola, melancholia with suicidal tendency is the most common.

In all the febrile diseases, disturbance of mental functions may appear as an initial delirium: (1) upon the onset of fever; (2) at the height of the fever; (3) after the disappearance of the fever, as a post-febrile exhaustion psychosis. In the initial delirium and in the febrile delirium, the theory is held "that the presence of certain toxalbumins, resulting from the high temperature and from the chemical action of the specific micro-organisms, combined with increased rapidity of circulation, cause the delirium. Berkley puts the matter tersely in a single sentence:

The over-setting of the mental functions is due to the virulence of the bacterial toxalbumins combined with high fever and deprivation of a sufficient nutrient supply to the cerebrum.

Regis, quoting Chardon's thesis of 1889, says:

Infectious diseases may cause insanity in three ways: (1) by the direct action of the microbes localised in the nervous centers; (2) by the action of the products secreted by the microbes; (3) by autointoxication by products not duly eliminated by the patient.

In the cases of post-febrile insanity, we have the condition of the patient characterised as exhaustion. The specific poison of the bacteria has been met and overcome either by tissue resistance or by the development of an antitoxin, and the patient, so far as the specific disease is concerned, is getting well, when after only a few hours of either excitement or confusion, or depression, the patient passes into actual insanity. The theories advanced to account for this have been the exhaustion theory.

Sterns, the medical superintendent of the Hartford Retreat, in his lectures at Yale Medical School, supports this theory of exhaustion. He argues that as patients are treated more and more on the plan of increased nutrition, patients with post-febrile insanity are less numerous than formerly.

Kraepelin, Berkley, and Regis believe that the intoxication and the inanition theory together are the important factors in the febrile and post-febrile psychoses.

The subject of toxins of insanity, as may be seen, covers a wide field, and one that has only lately been studied. The present writers of textbooks on insanity all refer to the subject and are more or less vigorous advocates of the theory of toxins as a cause of insanity.

As a summary, I quote from Peterson and from Bannister as the last American writers on the subject. Peterson says:

> The more mysterious poisons produced by disease in various parts of the body, by fermenting or putrefying substances in the alimentary tract, and by some of the infectious fevers, have only of late taken an important place in the etiology of the psychoses. We do not yet know how frequently autointoxication from absorption of intestinal poisons determines insanity, but the facts so far collected point to the origin of a considerable number of cases from this cause.

In Brower and Bannister we read:

> Intestinal autointoxication is one of the best established etiologic factors of insanity. In the severer infectious disorders, mental derangement is in many cases, and to a large extent, due to the toxins.

Thus, it may be seen the theory of toxins of insanity is one which is winning adherents. Its close connection with so many of the general diseases makes it of special interest to the general practitioner and specialist alike.

525 Delaware Avenue

